# Establishment of a Real-Time Fluorescence Isothermal Recombinase-Aided Amplification Method for the Detection of H9 Avian Influenza Virus

**DOI:** 10.3390/vetsci11090411

**Published:** 2024-09-05

**Authors:** Yuxin Zhang, Cheng Zhang, Jiaqi Li, Yejin Yang, Ligong Chen, Heng Wang, Zitong Yang, Mingda Zhang, Huan Cui, Shishan Dong

**Affiliations:** College of Veterinary Medicine, Hebei Agricultural University, Baoding 071000, China; yuxzhang2000@163.com (Y.Z.); chengzhang1349@hebau.edu.cn (C.Z.); ljq15027874688@163.com (J.L.); yejin317@outlook.com (Y.Y.); clg01@163.com (L.C.); wangheng946@163.com (H.W.); yang5241225@163.com (Z.Y.); zmd19832229138@163.com (M.Z.)

**Keywords:** H9, AIV, RT–RAA, visualization, rapid detection

## Abstract

**Simple Summary:**

This study successfully established a rapid and visual real-time fluorescence reverse transcription recombinase-aided isothermal amplification (RT–RAA) method for the detection of H9 subtype of avian influenza virus (AIV). We designed primers and probes with high specificity to ensure the accuracy and reliability of the detection method. The results show that this method had no cross-reaction with other influenza viruses, and its detection accuracy was highly consistent with that of real-time fluorescence quantitative PCR (RT–qPCR). Additionally, the amplification products of this method can be directly observed with the naked eye through a portable blue light instrument. This feature significantly enhances the intuition of the detection results, enabling non-professionals to quickly judge the detection results, thereby greatly improving the ease of use and popularity of the detection method. In resource-limited environments, this intuitive and rapid detection method is particularly important for the timely discovery and control of H9 AIVs.

**Abstract:**

The H9 subtype of avian influenza virus (AIV) has been characterized by its rapid spread, wide range of prevalence, and continuous evolution in recent years, leading to an increasing ability for cross-species transmission. This not only severely impacts the economic benefits of the aquaculture industry, but also poses a significant threat to human health. Therefore, developing a rapid and sensitive detection method is crucial for the timely diagnosis and prevention of H9 AIVs. In this study, a real-time fluorescent reverse transcription recombinase-aided isothermal amplification (RT–RAA) technique targeting the hemagglutinin (HA) of H9 AIVs was established. This technique can be used for detection in just 30 min at a constant temperature of 42 °C, and it exhibits good specificity without cross-reactivity with other viruses. Sensitivity tests revealed that the detection limit of RT–RAA was 163 copies per reaction, and the visual detection limit was 1759 copies per reaction at a 95% confidence interval, both of which are capable of detecting low concentrations of standards. Furthermore, RT–RAA was applied to detect 155 clinical samples, and compared to real-time fluorescent quantitative PCR (RT–qPCR), RT–RAA demonstrated high accuracy, with a specificity of 100% and a kappa value of 0.96, indicating good correlation. Additionally, with the assistance of a portable blue imaging device, we can visually observe the amplification products, greatly facilitating rapid detection in resource-limited environments. The RT–RAA detection method developed in this study does not require expensive equipment or highly skilled staff, making it beneficial for the accurate and low-cost detection of H9 AIVs.

## 1. Introduction

H9N2 is a low-pathogenic avian influenza virus (AIV) that has been widely spread globally in recent years, causing significant economic losses to the poultry industry. H9N2 AIV belongs to the Orthomyxoviridae family and the influenza virus A genus. Its viral particles are spherical, with diameters ranging from approximately 80 to 120 nanometers [1]. These viral particles possess two critical structures on their surface: hemagglutinin (HA) and neuraminidase (NA) [2]. Notably, HA may undergo recombination or mutation under certain circumstances, increasing the complexity of the pathogenic ecology of H9N2 AIV [3]. In the early 1990s, China experienced its first outbreak of H9N2 AIV. The epidemic rapidly spread in a short period, affecting up to 17 chicken farms and two bird habitats [4]. Current research indicates that the transmission rate of H9N2 AIV is closely linked to the migratory activities of migratory birds, and its seasonal pattern of incidence aligns with the migration cycle of wild birds. The virus spreads to different environments through the feces, saliva, and other excretions of migratory birds, continuously expanding its infection range and accelerating its transmission speed [5]. In recent years, H9N2 AIV has continued to evolve, increasing the complexity of its pathogenic ecology. In Egypt, the H9N2 strain evolved, enhancing the ability of the virus to spread from birds to humans [6]. Importantly, H9N2 AIV can not only be directly transmitted from birds to humans, but can also indirectly infect humans through intermediate hosts such as pigs and dogs [7]. Additionally, it can spread through aerosols or direct contact via media such as droplets, dust, feed, and water. Our previous research revealed that H9N2 AIVs can cross host barriers and infect birds, other mammals, and even humans through environmental transmission, resulting in cross-species transmission. This undoubtedly has a profound negative impact on the production and economic benefits of the aquaculture industry and human health [8]. Although the H9N2 subtype is currently classified as a low-pathogenic avian influenza virus, once the virus mutates or undergoes genetic recombination with other highly pathogenic viruses, it may produce a more threatening new virus strain. Therefore, we must maintain a high level of attention and focus on H9N2 AIVs to effectively address this public health challenge.

Currently, detection methods for H9N2 AIV primarily include quantitative real-time PCR, loop-mediated isothermal amplification (LAMP), serological testing, immunoassay technology, and gene chip technology [9,10]. According to research on the use of RT–qPCR for H9N2 detection, while the technology exhibits high sensitivity, its amplification process requires multiple temperature gradients, demanding advanced instrumentation and high costs [11]. On the other hand, although the LAMP technology used for detecting H9 subtype avian influenza virus requires only isothermal amplification without complex instrumentation, its primer design process is relatively complicated, necessitating the design of at least two pairs of primers [12]. Furthermore, a study established a double antibody sandwich enzyme-linked immunosorbent assay (DAS-ELISA) method to detect H9-subtype viral antigens [13]. Despite its high sensitivity, this technology can still produce false positive or false negative test results due to cross-reactions, nonspecific binding, or sample interference when processing complex samples. Additionally, gene chip technology can also be used for AIV detection, offering high detection efficiency and the ability to simultaneously test hundreds of samples [14]. However, the development of gene chips incurs high costs and requires highly skilled operators, making them unsuitable for use in basic laboratories. Therefore, there is an urgent need to develop a simple, rapid, and sensitive detection method for the on-site rapid detection of H9N2 AIV.

The reverse transcription–recombinase-aided amplification (RT–RAA) method has been successfully applied for the detection of various viruses, such as Newcastle disease virus and porcine reproductive and respiratory syndrome virus. This method, characterized by its efficiency and sensitivity, has played a significant role in areas such as animal disease prevention and monitoring. Compared to traditional molecular detection methods, RT–RAA does not require complex amplification techniques. It can quickly complete the detection process within 5 to 30 min at a constant temperature of 37 to 42 °C, significantly reducing the detection time [15]. Additionally, RT–RAA reagents exist in the form of lyophilized powder, eliminating the need for cold chain storage and facilitating transportation to monitoring sites and diagnostic scenes [16]. Therefore, this study successfully constructed a visual RT–RAA detection method targeting the HA gene of H9N2. With the assistance of a portable blue light imager, amplification results can be visualized intuitively and conveniently. Compared to the traditional RT–qPCR method, the RT–RAA detection method proposed in this study not only reduces the dependence on expensive equipment, but also lowers the requirement for technicians. This method provides strong technical support for the rapid screening and prevention of the H9N2 virus, making it suitable for the rapid detection of H9N2 AIV in resource-limited epidemic sites.

## 2. Materials and Methods

### 2.1. Source of Viruses and Clinical Samples

In this study, the virus strain A/chicken/Hebei/BD17/2023 (H9N2) was used. In 2024, 155 clinical samples suspected of being infected with AIV were collected from different regions of Hebei Province. These samples included trachea, lung, oropharyngeal and cloacal swabs. The details of the sample are shown in Supplementary Table S1.

### 2.2. Design of the RT–RAA Primer and Probe

The H9N2 HA gene sequence was downloaded from GenBank and aligned using DNASTAR software (version 11.1). Primers and probes were designed for conserved sequence regions using SnapGene software (version 4.3.6). Since RT–RAA requires the use of forward and reverse primers to find appropriate target sites on DNA double strands, five pairs of primers were designed to pinpoint the precise locations of the target sites, as detailed in Table 1. The optimal primers and probes were screened according to methods described in our previous study [17]. Specifically, a forward primer was randomly selected to screen for the best reverse primer, which was subsequently used to screen different forward primers, ultimately yielding the best primer pair. All primers and probes were synthesized by Shanghai Sangon (Shanghai, China).

### 2.3. Nucleic Acid Extraction

According to the instructions of the Qiagen RNA isolation kit (Hilden, Germany), RNA was extracted from A/swine/Hebei/SD17/2023 (H1N1), A/swine/Hebei/ZC55/2023 (H3N2), A/chicken/Hebei/CY59/2023 (H3N8), A/chicken/Hebei/HB777/2006 (H5N1), A/chicken/Hebei/CK05/2019 (H5N6), A/quail/Hebei/CH06-07/2018 (H7N9), A/chicken/Hebei/BD17/2019 (H9N2), Infectious Laryngotracheitis Virus (ILTV), Infectious bronchitis virus (IBV), Newcastle disease virus (NDV), Fowl adenovirus-4 (FAdV-4), Duck tembusu virus (DTMUV), Infectious bursal disease virus (IBDV), Goose astrovirus-1 (GastV-1), Goose astrovirus-2 (GastV-2) and 155 clinical samples suspected of AIV infection. The extracted RNA was then eluted with 50 μL of nuclease-free water and stored at −80 °C.

### 2.4. RT–RAA Amplification and RT–qPCR Assay

The RNA constant-temperature rapid amplification kit was purchased from AmpFuture Biotechnology Co., Ltd. (Weifang, China), and amplification was performed according to the user manual. A 25 μL reaction mixture was prepared, consisting of 14.7 μL of Buffer A, 4.75 μL of RNase-free water, 1.0 μL of forward primer (10 μM), 1.0 μL of reverse primer (10 μM), 0.3 μL of probe (10 μM), 2.0 μL of nucleic acid template, and 1.25 μL of Buffer B. The reaction tubes were then placed in a Gentier 96E qPCR machine (Tianlong, Xi’an, China) to monitor the fluorescent signal (at 42 °C for 30 min, with 1 cycle per minute). Additionally, a portable blue light imager (TIANGEN, Beijing, China) with an excitation wavelength of 480 nm was used for the visualization of the RT–RAA amplification products.

The RT–qPCR kit was obtained from Novizan Biotechnology (Q223, Nanjing, China), and amplification was performed according to the user manual utilizing primers and probes previously reported in a previous study [18]. The 25 μL reaction mixture consisted of 12.5 μL of 2× one step U+ Mix, 1.5 μL of one step U+ Enzyme Mix, 1.0 μL of forward primer (10 μM), 1.0 μL of reverse primer (10 μM), 0.5 μL of probe (10 μM), 2.0 μL of RNA template, and 6.5 μL of RNase-free water. The reaction tubes were placed in a Gentier 96E qPCR machine (Tianlong, Xi’an, China) to monitor fluorescent signals with the following program settings: 37 °C for 2 min, followed by 40 cycles at 95 °C for 5 min, 95 °C for 10 s, and 60 °C for 30 s.

### 2.5. Specific Analysis

The RNAs of H1N1, H3N2, H3N8, H5N1, H5N6, H7N9, H9N2, ILTV, IBV, NDV, FAdV-4, DTMUV, IBDV, GastV-1 and GastV-2 were used as templates for RT–RAA detection using the optimal primer pairs and probes selected above to verify their specificity.

### 2.6. Sensitivity Analysis

The PMD-18-T-HA plasmid of H9 AIV stored in the laboratory was used as a template to test the sensitivity of RT–RAA. The PMD-18-T-HA plasmid was diluted in a 10-fold gradient using ddH_2_O, with concentrations ranging from 1 × 10^5^ to 1 × 10^0^ copies per 2 µL. These dilutions were used as templates, ddH_2_O was used as a negative control, and eight replicates were used for each concentration. The optimized RT–RAA detection method was utilized to examine the sensitivity and evaluate the detection limit. Moreover, RT–qPCR was employed for parallel detection using the same templates to verify the accuracy of RT–RAA sensitivity. Finally, the data were analyzed using Statistical Product and Service Solutions (SPSS) software (version 24.0), applying probit regression.

### 2.7. Repeatability Analysis

The intra- and inter-group reproducibility of the RT-RAA method was tested using PMD-18-T-HA plasmid at concentrations of 10^7^, 10^5^, and 10^3^ copies per reaction and H9N2 virus nucleic acid as templates. To accurately evaluate its stability, the experiments were repeated three times within the same run and independently performed on three different days. The coefficient of variation (CV) of the threshold time was calculated for both intra-group and inter-group comparisons, and the reproducibility of RT–RAA was assessed based on these calculations.

### 2.8. Clinical Sample Testing

In total, 73 clinical samples suspected of having AIV were tested using the optimized RT–RAA method. To compare its accuracy, the same clinical samples were simultaneously tested using the RT–qPCR method, and the consistency of the results derived from the two detection methods was compared.

### 2.9. Statistical Analysis

SPSS software (version 24.0) was used to perform probit regression analysis on the data at a 95% probability level to determine the amplification limit. The kappa statistical method was used to compare the correlation between the RT–RAA and RT–qPCR results.

## 3. Results

### 3.1. Optimal Primer Screening for RT–RAA

**Figure 1:**
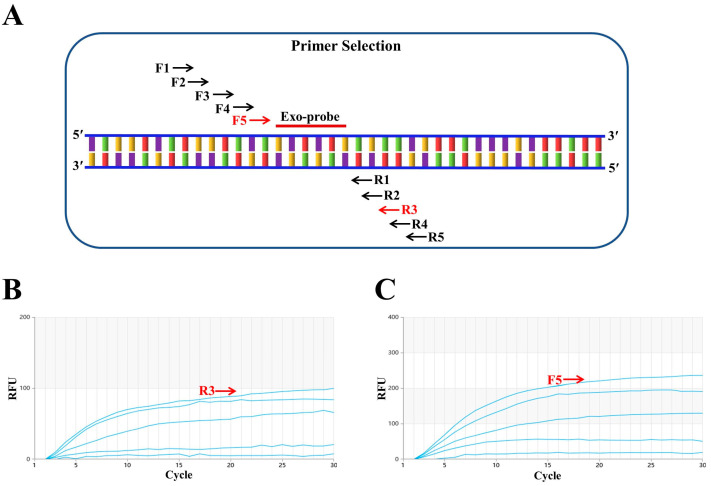
Screening of optimal primers. (**A**) F1, F2, F3, F4 and F5 represent upstream primers, while R1, R2, R3, R4 and R5 are downstream primers. F5 and R3, marked in red, indicate the optimal primer pair positions. Exo-probe refers to the exo probe. (**B**) Downstream primer screening results. The upstream primer F1 was randomly fixed, and among the five downstream primers screened, R3 performed the best. (**C**) Upstream primer screening results. With the best downstream primer R3 fixed, five upstream primers were evaluated, and F5 proved to be the most effective.

**Figure 2:**
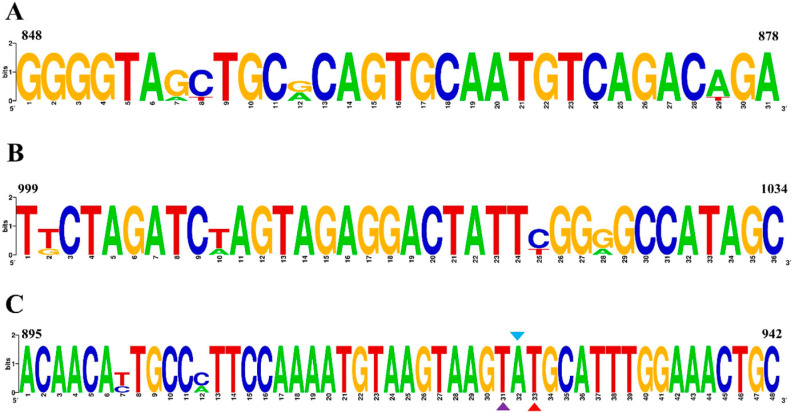
RT–RAA primers and probe. (**A**) Primer sequence of the optimal upstream primer F5 (F848-878). (**B**) Primer sequence of the optimal downstream primer R3 (R999-1034). (**C**) The sequence of probe p895-942. Within p895-942, the two T residues labeled with fluorophore (FAM) and quencher (BHQ1) are marked with purple and red triangles, respectively. The blue triangle represents the THF (tetrahydrofuran) spacer.

First, a conserved region close to the 5′ end was selected to design the probe (p895-942). Focusing on the ideal probe p895-942, five upstream primers (F1, F2, F3, F4 and F5, as shown in Figure 1A) and five downstream primers (R1, R2, R3, R4 and R5, as shown in Figure 1A) were designed. In the initial screening, the upstream primer F1 was randomly fixed and amplified with each of the five downstream primers. The fluorescence intensity was highest when the samples were paired with R3 (Figure 1B). Subsequently, the downstream primer R3 was fixed to screen the upstream primers, and the fluorescence intensity was found to be highest when R3 was paired with F5 (Figure 1C). Therefore, the combination of primers F5 and R3 with probe p895-942 was ultimately used for the RT–RAA detection of the H9N2 HA gene. Figure 2 illustrates the specific base sequences of the probe and the optimal upstream and downstream primers.

### 3.2. Specific Analysis of RAA

**Figure 3:**
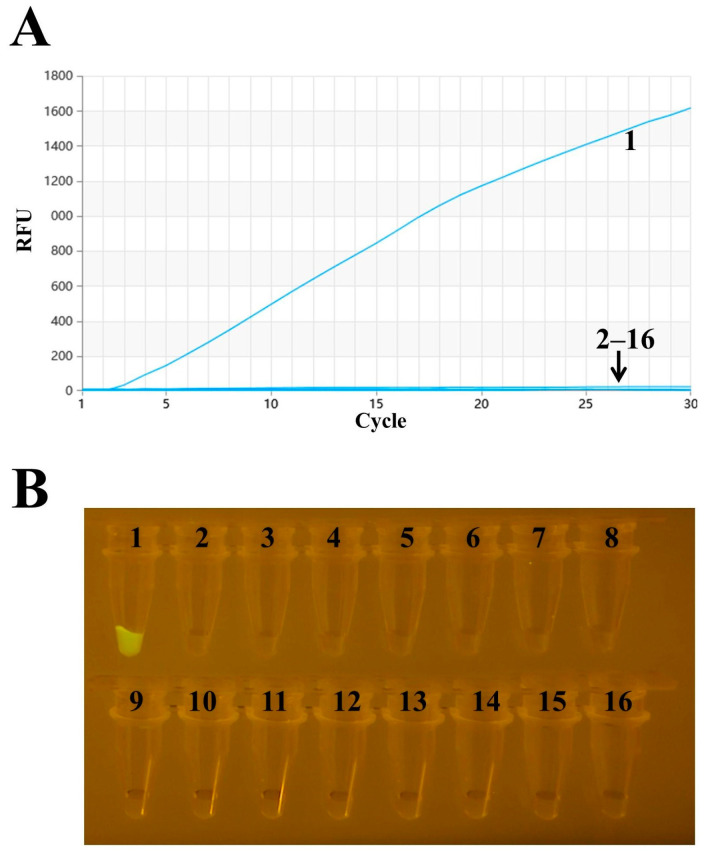
Specificity analysis of H9 AIV. (**A**) RT–RAA detection results. (**B**) Observation of RT–RAA amplification products using a portable blue imaging device. Numbers 1–16 represent H9N2, H1N1, H3N2, H3N8, H5N1, H5N6, H7N9, ILTV, IBV, NDV, FAdV-4, DTMUV, IBDV, GastV-1, GastV-2 and the negative control, respectively.

The specificity was tested using RT–RAA, with ddH2O serving as a negative control. The results indicate that only H9N2 was positive, while there was no cross-reaction with other viruses, with H1N1, H5N1, H5N6, H7N9, ILTV, IBV, NDV, FAdV-4, DTMUV, IBDV, GastV-1 and GastV-2 demonstrating good specificity (Figure 3). The amplified products were visually observed using a portable blue imaging device, and the results were consistent with those obtained from RT–RAA detection, thus confirming the specificity of the RT–RAA detection method.

### 3.3. Sensitivity Analysis of RAA

**Figure 4:**
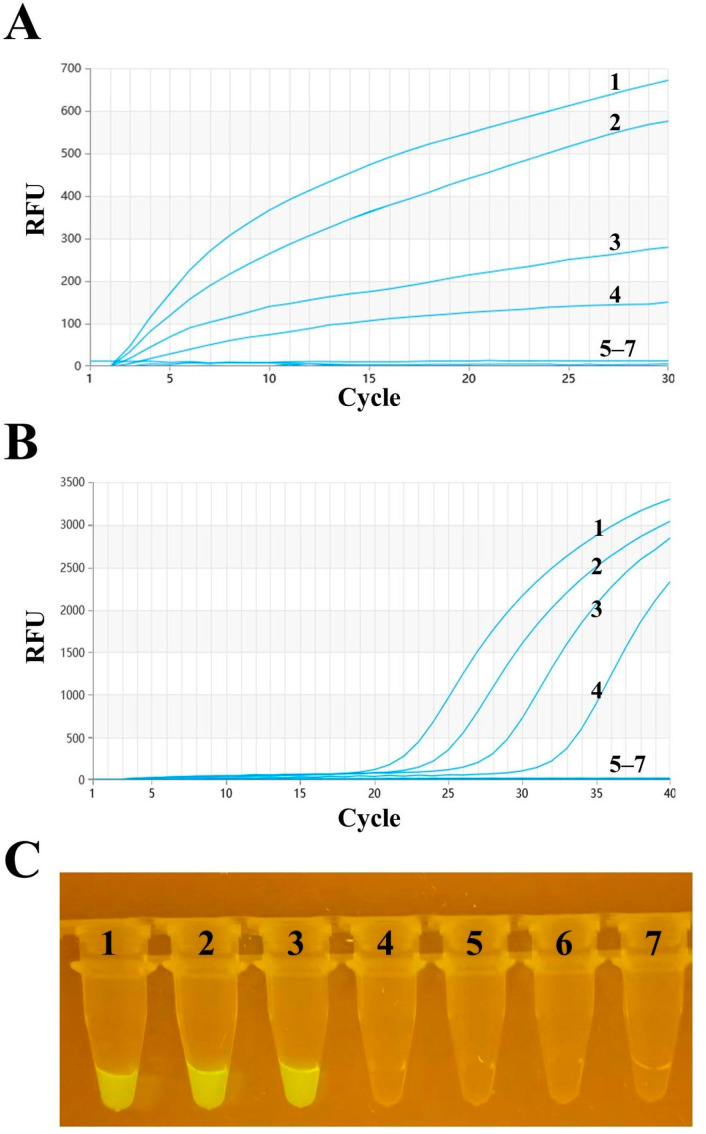
Sensitivity analysis of H9 AIV. (**A**) Detection sensitivity of the RT–RAA method. Numbers 1–6 represent templates with plasmid dilution concentrations ranging from 1 × 10^5^ to 1 × 10^0^ copies per reaction. Number 7 represents the negative control. (**B**) RT–qPCR detection sensitivity. The templates and their order are the same as those used in RT–RAA. (**C**) Observation of RT–RAA amplification products using a portable blue imaging device.

The optimized RT–RAA method was used to detect the 10-fold serially diluted standard PMD-18-T-HA plasmid, with dilution concentrations ranging from 1 × 10^5^ to 1 × 10^0^ copies per reaction. A parallel experiment using the same template was conducted with RT–qPCR to compare the detection results of the two methods. According to Figure 4A, the limit of detection (LOD) for RT–RAA was 163 copies per reaction at a 95% confidence interval. Figure 4B shows that the LOD for RT–qPCR was 38 copies per reaction at a 95% confidence interval. Additionally, observation through a portable blue imaging device revealed a visual LOD of 1759 copies per reaction at a 95% confidence interval. Upon comparison, it was found that these methods could detect relatively low concentrations of the standard, demonstrating good sensitivity.

### 3.4. RT–RAA Repeatability Analysis

To evaluate the reproducibility of the RT-RAA method, a standard product with concentrations diluted in gradients of 10^7^, 10^5^ and 10^3^ copies/reaction and an H9N2 virus nucleic acid were used as a template for detection. Intra-group reproducibility was tested by repeating the assay three times within the same run, while inter-group reproducibility was evaluated by performing three independent runs on different days. As shown in Table 2, the intra-group coefficient of variation ranged from 4.01% to 5.02%, and the inter-group coefficient of variation ranged from 3.53% to 6.67%. The low levels of both intra-group and inter-group variation coefficients indicate that the RT-RAA detection technology has good reproducibility and stability.

### 3.5. Clinical Sample Testing

To evaluate the accuracy of the RT–RAA method used for detecting H9 AIV, 73 clinical samples were simultaneously tested using both the optimized RT–RAA and RT–qPCR detection methods. As shown in Table 3, RT–RAA detected 83 positive samples and 70 negative samples, with a sensitivity of 97.65%, a specificity of 100%, a kappa value of 0.96 (*p* < 0.001), and an overall accuracy of 98.70% (153/155). The results of visual RT–RAA detection show that 78 positive samples and 70 negative samples were detected, with a sensitivity of 91.76%, specificity of 100%, and kappa value of 0.91 (*p* < 0.001), demonstrating good correlation and an overall accuracy of 95.48% (148/155). In summary, the visual RT–RAA detection method can quickly and accurately detect 73 clinical samples.

## 4. Discussion

Since the first detection of H9N2 avian influenza virus (AIV) in turkeys in 1966, the virus has spread widely across the globe [19]. When birds are infected with H9N2 AIV, they mainly exhibit clinical symptoms such as growth retardation and decreased egg production, causing significant economic losses to the poultry industry [20]. Currently, many family farms adopt free-range or mixed grazing modes for raising poultry. However, this approach may expose poultry to other domestic birds or wild waterfowl during daily activities. Notably, these healthy birds may carry H9N2 AIV, increasing the risk of widespread transmission of the virus among birds [21]. The genome of H9N2 consists of eight RNA segments, facilitating the exchange of gene fragments through recombination and resulting in the emergence of novel recombinant strains. Some of these recombinant strains can cross species barriers, which has significant implications for virus evolution and cross-species transmission [22,23]. In recent years, there have been increasing cases of H9N2 transmission to humans. As of the end of April 2024, 127 cases of human infection with H9N2 avian flu have been reported globally, including four cases of infection in China in March 2024. The severity of infection is related to the health status of the infected individual, and the number of infected patients is greater in winter and spring than in summer and autumn [24]. As the ecological complexity of H9N2 pathogens increases, the harmful effects of H9N2 on birds and other mammals gradually increase. Efficient and sensitive AIV detection methods, which limit further virus spread and reduce potential harm to the aquaculture industry and human health, are crucial for rapid virus detection.

The detection method for H9N2 AIV has always been a focus of avian influenza research. Although there have been many studies on the detection of H9N2 AIV in the past, they all have certain limitations. Among them, RT–qPCR is a widely used technique in the field of molecular biology, and previous studies have used this method to detect H9N2 AIV [25]. Although RT–qPCR can directly detect viral nucleic acid with good specificity, its operation is relatively complex and requires expensive equipment and instruments. Additionally, fluorescent immunoassay chromatographic test strips (FICTs) have been established to detect hemagglutinin of the H9 subtype avian influenza virus in feces [26]. However, the europium nano-like compounds used in this method have strict storage requirements. Especially in damp environments, their performance can significantly decline, which may directly affect the accuracy of test results. Therefore, in practical applications, special attention needs to be paid to storage conditions to ensure the reliability of the test results.

RT–RAA technology has broad application prospects and market demand for use in avian influenza detection. Successful studies have already applied this technology to avian influenza detection, demonstrating its high accuracy in pathogen detection. Some research has combined RT–RAA with lateral flow immunoassay (LFD) to detect H9N2 AIV, and the results indicate the specific amplification of H9N2 under constant temperature conditions [27]. Additionally, other studies have used RT–RAA to amplify the H7 subtype of avian influenza virus. By designing specific primers and probes, the accurate detection of the target virus has been achieved without cross-reactivity with other common viruses, exhibiting good specificity [15]. The RT–RAA method is easy to perform and highly specific, requiring no complex equipment or specialized technical personnel. This makes the method advantageous for early disease diagnosis and rapid on-site testing. However, while RT–RAA technology provides fast and accurate detection, there is still a need to improve the intuitiveness of RT–RAA test results in practical applications.

In this study, a visual RT–RAA method targeting the conserved region of the HA gene was developed to detect H9N2 AIV. The detection process could be completed in just 30 min at a constant temperature of 42 °C. Additionally, a portable blue imaging device was utilized for visual observation of the amplification results, eliminating the need for expensive reagents and skilled technicians. This achieved the visualization of amplification products, facilitating on-site detection in resource-limited settings. Initially, the conserved region was identified through sequence alignment of the H9N2 HA gene sequences retrieved from GenBank. Based on this, an exo probe was designed with a length ranging from 46 to 52 bp. Five upstream and downstream primers were designed around the probe. By fixing one upstream primer to screen downstream primers and then pairing the best downstream primer with each of the five upstream primers, the optimal primer combination F5 + R3 was selected. This screening method was consistent with previous approaches [17]. RT–RAA was used to simultaneously detect H9N2 along with ILTV, IBV, NDV, FAdV-4, DTMUV, IBDV, GastV-1, GastV-2, H1N1, H3N2, H3N8, H5N1, H5N6, and H7N9. The results show that only H9N2 tested positive, demonstrating the high specificity of the RT–RAA detection method and its reliability. The limit of detection for RT–RAA was 163 copies per reaction at a 95% confidence interval, with sensitivity comparable to that of the previously reported RT–RPA (recombinase polymerase amplification) and LFD detection method [28]. However, the RT–RAA method established in this study did not require the use of LFDs. Instead, it utilizes a portable blue imaging device to achieve the rapid visual detection of amplification products, making it more suitable for low-cost on-site diagnosis. Furthermore, 155 clinical samples were tested using both RT–RAA and RT–qPCR detection methods. RT–RAA identified 83 positive samples, while RT–qPCR detected 85 positive samples. The accuracy of the RT–RAA detection method was 97.65% (153/155), with a kappa value of 0.96 (*p* < 0.001), indicating the good accuracy of the test results. Notably, visual RT–RAA detected 78 positive samples with an accuracy of 91.76% (148/155). This suggests that the visual RT–RAA detection method can be effectively used for the clinical diagnosis of H9N2 AIV.

Despite the promising results, this study on H9 RT-RAA exhibits several limitations. Firstly, a notable challenge is related to achieving efficient on-site sample pre-treatment, especially nucleic acid extraction, which can be laborious and may require specialized equipment and trained personnel, potentially limiting its accessibility across diverse settings. Secondly, another constraint lies in the need for multi-target detection with high sensitivity and specificity. Currently, RT-RAA may lack the capability to detect multiple targets simultaneously with the desired accuracy, possibly restricting its application in certain scenarios. It is essential to recognize that without addressing these challenges, the application scope of RT-RAA may remain constrained. Therefore, further research and development are imperative to overcome these limitations and expand the potential applications of RT-RAA.

## 5. Conclusions

The RT–RAA detection method established in this study for H9N2 AIV has unique advantages in clinical diagnosis compared to traditional qPCR detection methods. First, RT–qPCR requires 1.5–2 h to complete amplification, whereas the RT–RAA detection method can be completed in just 30 min at a constant temperature of 42 °C, without requiring complex equipment. This makes it suitable for on-site diagnosis in resource-limited and time-critical situations. Second, based on the specificity and sensitivity demonstrated by the RT–RAA detection method developed in this study, it can serve as a highly reliable diagnostic tool for detecting H9N2 AIV. Specifically, the results of this method can be directly observed with the naked eye under a portable blue imaging device, and the detection results are relatively accurate, making it suitable for the rapid screening of H9N2 AIV at the grassroots level. With the frequent occurrence of H9N2 avian influenza and increasing public concern about food safety and public health security, visual RT–RAA detection technology is expected to play a significant role in the early detection, rapid diagnosis, and disease control of H9N2 AIV, providing a more efficient and convenient detection method for the prevention and control of H9N2 subtype avian influenza.

## Figures and Tables

**Table 1:** The primers and probes used in H9 AIV real-time RT–RAA assays.

Primers/Probe	Sequences (5′→3′)	Position
H9-F1	AGAATAAAATCTGATGGGAATCTAATAGCTC	754–784
H9-F2	ACATTCTTTCAGGAGAGAGCCATGGAAGAA	797–826
H9-F3	TTTCAGGAGAGAGCCATGGAAGAATTCTGAG	803–833
H9-F4	GAAGAATTCTGAGGACTGATCTAAAAAGGG	821–850
H9-F5	GGGGTAGCTGCGCAGTGCAATGTCAGACAGA	848–878
H9-R1	TGCAAGTTTGAGACTCTTTATTCCAATGTA	949–978
H9-R2	TCAGACCAACTGCAAGTTTGAGACTCTTTA	959–988
H9-R3	TTCTAGATCTAGTAGAGGACTATTCGGGGCCATAGC	999–1034
H9-R4	CATACCAACCAGCAACTAGTCCTGACCAACCTCCT	1047–1081
H9-R5	CTGCCATACCAACCCCCTGGTCATTTGAATGCTGAA	1085–1120
H9-probe	ACAACATTGCCCTTCCAAAATGTAAGTAAG(FAM-dT)(THF)(BHQ1-dT) GCATTTGGAAACTGC[C3-spacer]	895–942

**Table 2:** Results of repeatability and reproducibility detected by real-time RAA assay.

Plasmids Concentration	Repeatability (Intra-Batch Assay)			Reproducibility (Inter-Batch Assay)		
	Mean	SD	CV (%)	Mean	SD	CV (%)
High (10^7^)	119.33	5.13	4.30	129.33	5.51	4.26
Medium (10^5^)	226.33	9.07	4.01	228.33	8.08	3.53
Low (10^3^)	369.33	18.56	5.02	373.67	24.91	6.67
H9N2 virus nucleic acid	253.67	11.37	4.48	252.33	12.50	4.96

Mean: average threshold times (seconds) of three independent real-time RAA reactions. SD: standard deviation. CV: coefficient of variation.

**Table 3:** Comparison of H9 AIV real-time RT–RAA with RT–qPCR in clinical samples.

Assay		RT–qPCR		Sensitivity	Specificity	Kappa
		Positive	Negative			
Real-time RT–RAA (Via real-time fluorescence read-out)	Positive	83	0	97.65%	100%	0.96
	Negative	2	70			
	Total (155)	85	70			
Real-time RT–RAA (Via visual detection)	Positive	78	0	91.76%	100%	0.91
	Negative	7	70			
	Total (155)	85	70			

## Data Availability

The original contributions presented in this study are included in the article. For further inquiries, please contact the corresponding authors.

## Supplementary Materials

The following supporting information can be downloaded at: https://www.mdpi.com/article/10.3390/vetsci11090411/s1, Table S1: List of the samples, the nucleic acid extraction quantification and the purity.
