# Supplementary material for: Establishment of a Real-Time Fluorescence Isothermal Recombinase-Aided Amplification Method for the Detection of H9 Avian Influenza Virus
Source: Vet Sci. 2024 Sep 5;11(9):411. doi: 10.3390/vetsci11090411 (PMC11436240; doi:10.3390/vetsci11090411)
Supplement: Supplementary file 1 [file vetsci-11-00411-s001.zip › vetsci-3102728-supplementary.pdf]

S1 Table. List of the samples, the nucleic acid extraction quantification and the purity

Sample ID	Sample type	Nucleic acid concentration (ng/μL)	A260/A280 Ratio	A260/A230 Ratio
1	trachea	126.76	1.996	2.295
2	trachea	174.96	2.019	2.177
3	trachea	120.28	2.006	2.346
4	lung	102.72	2.032	2.398
5	lung	111.68	2.051	2.237
6	lung	83.600	2.017	2.312
7	lung	182.52	2.020	2.387
8	lung	56.520	2.045	2.424
9	lung	104.28	1.975	2.434
10	oropharyngeal swab	196.56	2.015	2.278
11	oropharyngeal swab	229.20	2.036	2.344
12	oropharyngeal swab	54.320	2.012	2.553
13	oropharyngeal swab	93.160	2.025	2.367
14	oropharyngeal swab	67.880	2.045	2.459
15	trachea	103.24	2.042	2.433
16	trachea	96.920	2.009	2.478
17	trachea	117.04	1.970	2.503
18	trachea	188.40	2.035	2.368
19	trachea	150.12	2.035	2.387
20	trachea	81.080	2.043	2.430
21	trachea	198.64	2.046	2.386
22	lung	211.56	2.041	2.369
23	lung	65.640	2.013	2.457
24	lung	120.72	2.034	2.343
25	lung	157.96	2.048	2.181
26	lung	117.40	2.037	2.254
27	cloacal swab	164.92	2.014	2.339
28	cloacal swab	198.16	2.036	2.358
29	cloacal swab	39.040	2.063	2.307
30	cloacal swab	157.12	2.058	2.404
31	cloacal swab	146.80	2.047	2.336
32	cloacal swab	133.20	2.028	2.396
33	oropharyngeal swab	204.36	2.042	2.373
34	oropharyngeal swab	231.60	2.084	2.099
35	oropharyngeal swab	203.24	2.088	2.027
36	trachea	159.44	2.072	2.025
37	trachea	67.720	2.023	2.132
38	trachea	140.44	2.001	2.063
39	trachea	131.88	2.066	2.144
40	lung	80.880	2.030	2.055
41	lung	66.800	2.098	2.012

42	lung	53.800	2.082	2.076
43	lung	117.28	2.099	2.015
44	lung	72.120	2.068	2.114
45	cloacal swab	148.84	2.090	2.162
46	cloacal swab	108.16	2.056	2.041
47	cloacal swab	186.20	2.094	2.128
48	lung	172.32	2.055	2.140
49	lung	143.64	2.087	2.132
50	oropharyngeal swab	135.96	2.089	2.140
51	oropharyngeal swab	47.880	2.071	2.071
52	oropharyngeal swab	94.440	2.004	2.106
53	oropharyngeal swab	82.880	2.010	2.152
54	lung	128.48	2.052	2.050
55	lung	96.240	2.090	2.168
56	oropharyngeal swab	130.88	2.092	2.118
57	oropharyngeal swab	331.08	2.082	2.085
58	oropharyngeal swab	90.920	2.089	2.095
59	oropharyngeal swab	152.92	2.068	2.061
60	trachea	73.240	2.044	2.008
61	trachea	187.96	2.076	2.120
62	trachea	271.44	2.076	2.109
63	lung	96.400	2.099	2.063
64	lung	21.480	2.026	2.050
65	lung	56.520	2.084	2.004
66	lung	101.40	2.100	2.010
67	lung	50.520	2.081	2.091
68	lung	108.72	2.100	2.197
69	oropharyngeal swab	42.240	2.087	2.047
70	oropharyngeal swab	42.080	2.087	2.011
71	oropharyngeal swab	64.800	2.074	2.007
72	oropharyngeal swab	64.800	2.074	2.007
73	oropharyngeal swab	149.24	2.098	2.017
74	trachea	81.400	2.074	2.015
75	trachea	53.160	2.067	2.020
76	trachea	211.88	2.083	2.046
77	trachea	68.200	2.062	2.087
78	trachea	71.320	2.066	2.042
79	trachea	97.480	2.097	2.121
80	trachea	123.48	2.100	2.083
81	cloacal swab	203.60	2.092	2.109

82	cloacal swab	129.96	2.063	2.021
83	cloacal swab	62.880	2.090	2.085
84	lung	117.40	2.087	2.110
85	lung	182.60	2.066	2.056
86	cloacal swab	221.72	2.093	2.095
87	cloacal swab	90.200	2.076	2.125
88	cloacal swab	110.32	2.067	2.035
89	lung	112.88	2.089	2.041
90	lung	92.800	2.088	2.193
91	cloacal swab	175.28	2.005	2.102
92	cloacal swab	75.520	2.050	2.098
93	cloacal swab	160.36	2.002	2.090
94	lung	86.720	2.065	2.197
95	lung	64.280	2.063	2.004
96	oropharyngeal swab	153.60	2.019	2.196
97	oropharyngeal swab	121.80	2.013	2.021
98	oropharyngeal swab	96.160	2.042	2.128
99	oropharyngeal swab	63.080	2.097	2.079
100	oropharyngeal swab	98.600	2.080	2.063
101	oropharyngeal swab	75.080	2.100	2.058
102	oropharyngeal swab	85.960	2.016	2.196
103	lung	98.960	2.100	2.003
104	lung	88.690	2.054	2.177
105	oropharyngeal swab	96.320	2.100	2.213
106	oropharyngeal swab	95.040	1.981	2.168
107	lung	81.240	2.091	2.227
108	lung	124.00	2.039	2.102
109	oropharyngeal swab	106.12	2.092	2.221
110	trachea	200.40	2.005	2.152
111	trachea	156.32	2.006	2.116
112	trachea	80.320	2.081	2.080
113	trachea	72.360	2.018	2.131
114	trachea	113.44	2.075	2.110
115	trachea	132.52	2.012	2.153
116	trachea	70.840	2.088	2.242
117	cloacal swab	83.880	2.085	2.174
118	trachea	83.440	2.033	2.257
119	trachea	96.040	2.011	2.046
120	trachea	63.920	2.062	2.207
121	trachea	57.960	2.006	2.082
122	trachea	83.960	2.085	2.266
123	trachea	87.280	2.095	2.025
124	trachea	135.88	2.100	2.138

125	cloacal swab	133.56	2.005	2.276
126	oropharyngeal swab	109.56	2.013	2.135
127	oropharyngeal swab	97.800	2.082	2.162
128	oropharyngeal swab	106.88	2.021	2.172
129	oropharyngeal swab	94.320	2.045	2.098
130	lung	96.520	1.863	2.229
131	lung	128.64	2.009	2.158
132	oropharyngeal swab	94.560	2.028	2.118
133	oropharyngeal swab	92.560	2.088	2.050
134	lung	105.960	2.010	2.025
135	lung	99.360	2.067	2.679
136	oropharyngeal swab	93.400	2.083	2.164
137	trachea	74.240	2.100	2.186
138	trachea	95.800	2.095	2.078
139	trachea	123.40	2.066	2.107
140	trachea	149.76	2.061	2.079
141	trachea	96.920	2.033	2.143
142	trachea	94.520	2.063	2.182
143	trachea	92.480	2.037	2.066
144	cloacal swab	137.82	2.002	2.069
145	trachea	126.60	2.095	2.183
146	trachea	90.840	2.067	2.104
147	trachea	95.600	2.050	2.187
148	trachea	96.520	2.007	2.125
149	trachea	73.480	2.066	2.114
150	cloacal swab	72.040	2.004	2.152
151	trachea	128.16	2.007	2.175
152	trachea	98.720	1.908	2.065
153	trachea	121.64	1.952	2.025
154	cloacal swab	167.16	2.100	2.068
155	trachea	96.64	2.001	2.095
